# Student assessment of teaching as a source of information about aspects of teaching quality in multiple subject domains: an application of multilevel bifactor structural equation modeling

**DOI:** 10.3389/fpsyg.2015.01550

**Published:** 2015-10-08

**Authors:** Ronny Scherer, Jan-Eric Gustafsson

**Affiliations:** ^1^Centre for Educational Measurement at the University of Oslo, Faculty of Educational Sciences, University of OsloOslo, Norway; ^2^Department of Education and Special Needs Education, Faculty of Education, University of GothenburgGothenburg, Sweden

**Keywords:** Bifactor structural equation modeling, cross-country differences, multilevel structural equation modeling, student achievement, teaching quality

## Abstract

Research on educational effectiveness most often uses student assessments of classroom instruction for measuring aspects of teaching quality. Given that crucial inferences on the success of education are based on these assessments, it is essential to ensure that they provide valid indicators. In this study, we illustrate the application of an innovative application of a multilevel bifactor structural equation model (ML-BFSEM) to examine the validity of student assessments. Analyzing a large-scale data set of 12,077 fourth-grade students in three countries (Finland, Norway, and Sweden), we find that (i) three aspects of teaching quality and subject domain factors can be established; (ii) metric and scalar invariance could be established for the ML-BFSEM approach across countries; and (iii) significant relations between students’ assessments of how easy the teacher is to understand and achievement in all subjects exist. In support of substantive research, we demonstrate a methodological approach for representing the complex nature of student assessments of teaching quality. We finally encourage substantive and methodological researchers to advance the ML-BFSEM.

## Introduction

Research on the effectiveness of teaching most often uses students’ assessments to study how the quality of teaching relates to educational outcomes such as student achievement and interest (Lüdtke et al., 2009; Mitchell et al., 2010; Fauth et al., 2014). Particularly in the context of educational large-scale assessments such as the Trends in International Mathematics and Science Study (TIMSS) and the Progress in International Reading Literacy Study (PIRLS), student assessments are incorporated for a number of reasons: (i) they are more objective measures than teachers’ self-ratings (Kunter and Baumert, 2006); (ii) they are easily accessible; and (iii) they provide valid information on the different aspects of teaching quality (Wagner et al., 2013). Against this background, researchers have focused on the appropriate use and modeling of these ratings, suggesting that they should be solely regarded as classroom-aggregated rather than as student-level variables (Lüdtke et al., 2009; Marsh et al., 2012). Besides taking a multilevel perspective on student assessments of teaching quality, research has also identified their complex nature. Indeed, these ratings are considered to be multidimensional comprising different aspects of teaching quality, subject-specific, and culturally sensitive (Marsh, 1991; Seidel and Shavelson, 2007; Klieme, 2013; Fauth et al., 2014). Although integrating the different perspectives on student assessments seems necessary to ensure valid representations of the construct, especially in cross-national studies, an integrated approach to describe their internal structure and the relations to external variables has rarely been taken. We suspect that this is most likely due to the complexity of the psychometric models that could potentially provide such an integrative view. This substantive need calls for a complex yet flexible modeling approach that integrates the multilevel structure, multidimensionality, subject specificity, and the potential existence of differing response styles across cultural contexts.

The current study presents a substantive–methodological synergism serving multiple purposes: making use of the TIMSS and PIRLS 2011 datasets for Finland, Norway, and Sweden (*N* = 12,077), we present a novel application of a multilevel bifactor structural equation model (ML-BFSEM) to describe students’ assessments of teaching quality and to stimulate further applications of this modeling approach. Moreover, we attempt to generate publicity of ML-BFSEM and create an awareness of its flexibility in representing the complex nature of students’ assessments (Reise, 2012). Substantively, our study is aimed at providing evidence on the internal and external validity of these assessments.

### Student Assessments of Teaching Quality

#### Level of Analysis

An important question that comes along with the use of student assessments concerns the appropriate level of analysis. Following Lüdtke et al. (2009), this decision depends on the research question posed. In particular, they distinguish between three types of questions that deal with: (i) the use of students’ perceptions of classroom instruction in order to describe the learning environment; (ii) the development of students’ motivation within learning environments; and (iii) the effects of teaching quality on student outcomes such as achievement. Whereas (i) mainly focuses on individual perceptions and the psychological climate of classrooms (Parker et al., 2003), (ii) and (iii) relate to typical questions of teaching effectiveness. Moreover, since large-scale educational assessments such as TIMSS are aimed at evaluating entire classrooms, schools, countries, or systems with respect to teaching quality, the decision for an appropriate level of analysis becomes even more crucial (Lüdtke et al., 2009; Marsh et al., 2011). Marsh et al. (2012) concluded that most researchers are interested in the effects of the classroom environment on educational outcomes and should, therefore, interpret student assessments as *classroom* rather than *individual* constructs. This recommendation is mainly based on the fact that student assessments rely on ratings of classrooms as clustering units (Morin et al., 2014). Marsh et al. (2012) consequently suggested using the aggregated student data at the classroom level, given that students’ individual perceptions of a classroom-level construct do not have a distinct meaning at the student level; yet, differences in student ratings are considered to be indicators of unreliability (Morin et al., 2014). As a consequence, it is necessary for the analysis to separate variation due to differences between teachers/classrooms on the one hand, and between students within classrooms on the other hand. One analytical approach that in principle at least is capable of dealing with these challenges is two-level structural equation modeling (Hox, 2013).

#### Multidimensional Structure

In substantive research on teaching quality, there is a consensus that the construct comprises a number of factors, each representing different aspects of classroom instruction (Marsh, 1991; Seidel and Shavelson, 2007; Creemers and Kyriakides, 2008). For instance, Klieme et al. (2009) suggested differentiating between at least three aspects, namely classroom management, cognitive activation, and teacher support. Other frameworks distinguish between even more factors or focus on alternative aspects of teaching (Abrami et al., 1990; Creemers and Kyriakides, 2008; Wagner et al., 2013). Interestingly, this multidimensionality of teaching quality has been supported empirically at both the student and the classroom level (Fauth et al., 2014). Against this background, we conclude that it is essential to account for the different aspects of teaching quality which may lead to a multidimensional structure.

#### Subject Specificity

Research that uses students’ assessments of teaching quality has indicated that these assessments are, at least to some extent, subject-specific (Klieme, 2013). For instance, Wagner et al. (2013) showed that, although the structure of teaching quality was invariant across subjects, the correlations between its factors in different subject domains were low. This subject specificity may have a number of reasons: (i) students’ views on teaching quality interact with their beliefs about the specific subject domain, leading to differences in ratings across subject domains (Buehl et al., 2002); (ii) given that students encounter different teachers across subject domains who may vary in their quality of teaching, differences in the student assessments may occur. It is, therefore, worthwhile accounting for these potential differences when modeling students’ assessments.

#### Cross-country Differences

One challenge in research involving participants from different cultural contexts is that questionnaire items may be responded to differently as a function of different ways of interpreting items and of communicating responses (Smith, 2011). Culturally related response styles form a systematic source of error variance in questionnaire responses, which may bias estimates of relations to other variables such as achievement. He and van de Vijver (2013) found both individual differences and differences between ethnic groups in several previously identified response styles (e.g., acquiescence, extremity, midpoint responding, and socially desirable responding). However, they also demonstrated that the different response styles identified a general response style factor, which was strongly related to a general factor of measures of personality. These results suggest that it is necessary to take into account differences in response style, and that this can be conducted by modeling response style as a general factor.

### The Present Study

**FIGURE 1:**
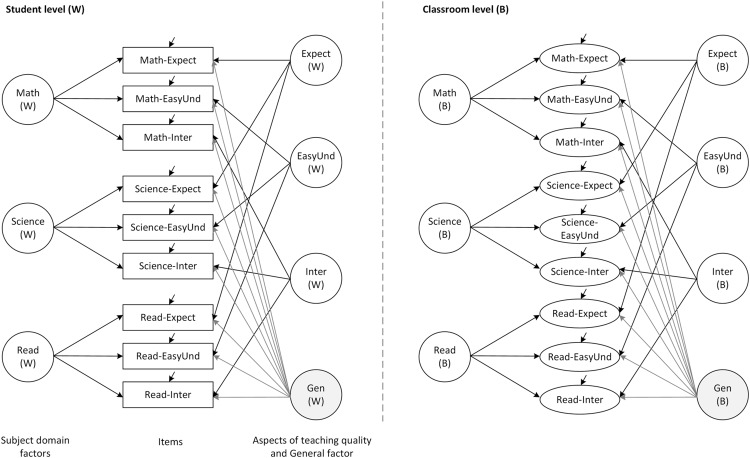
**Hypothesized multilevel bifactor structural equation model (ML-BFSEM) of student assessments.** Math, Mathematics; Read, Reading; Expect, Teacher expectations; EasyUnd, Easy to understand; Inter, Interest; Gen, General factor.

Taken together, our review of substantive research revealed that student assessments are of multilevel, multidimensional, subject-specific, and culturally sensitive nature. One approach that is capable of addressing this complex nature refers to multilevel bifactor structural equation modeling. For both the student and the classroom level, a number of factors can be specified that represent the aspects of teaching quality on the one hand, and the different subject domains on the other hand. In addition to these factors, a general factor captures students’ general response styles in the assessments of teaching quality. The resulting bifactor model contains a multitrait–multimethod structure, in which the teaching aspects are considered to be the traits and the subject domains represent the different methods (Eid et al., 2008; Castro-Schilo et al., 2013; Geiser et al., 2015). **Figure 1** presents the hypothesized multilevel structure for the student and the classroom level. In light of our considerations, we are aimed at illustrating the application of such a modeling approach that integrates the different characteristics of teaching quality by posing three research questions:

(1) To what extent does the ML-BFSEM represent the structure of student assessments of teaching quality?
(2) To what extent does the ML-BFSEM represent a measurement model that is invariant across three Nordic countries (Finland, Norway, and Sweden)?
(3) How do students’ assessments of aspects of teaching quality relate to their achievement in mathematics, science, and reading in the ML-BFSEM?

We notice that the first and second research questions are concerned with the internal validity of student assessments, whereas the third question addresses their external validity (Messick, 1995).

## Materials and Methods

### Sample and Procedure

The data were retrieved from the TIMSS and the PIRLS 2011. In particular, the sample comprised the large-scale data sets of Finland (*n*_FIN_ = 4,541), Norway (*n*_NOR_ = 3,054), and Sweden (*n*_SWE_ = 4,482). In total, *N* = 12,077 fourth-grade students in 715 classrooms and 416 schools (age: *M* = 10.5, *SD* = 0.6, Min = 8.4 years, and Max = 13.3 years; 49.2% girls; on average, 16.8 students per school) took a questionnaire on motivational, background, and teaching-related variables, and worked on performance tests in mathematics, science, and reading. TIMSS and PIRLS were administered jointly in 2011 for grade level four, such that the students who worked on tests and questionnaires in both studies could be identified (Martin and Mullis, 2013). These students were included in the analyses.

### Measures

#### Student Assessments of Teaching Quality

As stated earlier, the construct of teaching quality is multifaceted and comprises a number of aspects. In TIMSS and PIRLS 2011, a limited number of these aspects were assessed, given the large amount of contextual variables in the background questionnaire. These aspects focused on teachers’ clarity of goal orientation and instruction, that is the degree to which teachers communicate clearly in the classroom (Ames, 1992), and teachers’ emotional support. TIMSS and PIRLS have tied their instructional assessments toward students’ engagement, leading to a number of scales that refer to their perceptions about the degree to which they feel that the teacher engages them (Mullis et al., 2012). In particular, students were asked to rate the extent to what they agree with the following items (from 1 = *I agree a lot* to 4 = *I disagree a lot*): ‘I know what my teacher expects me to do’ (*Expect*), ‘My teacher is easy to understand’ (*EasyUnd*), and ‘I am interested in what my teacher says’ (*Inter*). These three items were administered for the three subject domains of mathematics, science, and reading, yielding nine items in total.

#### Student Achievement in Mathematics, Science, and Reading

In TIMSS 2011, students’ achievement in mathematics and science was assessed by tests that comprises items of different cognitive domains (i.e., *Knowing, Applying*, and *Reasoning*) and subject-specific contents domains (e.g., *Number, Earth Science*; Mullis et al., 2009b). Since 175 mathematics and 172 science items were used, a rotated-booklet design was implemented to reduce the number of items a single student had to work on. As a consequence, TIMSS 2011 provided a set of five plausible values for both mathematics and science achievement (Foy et al., 2013). These values are available for the overall achievement but also for the content and cognitive domains. In PIRLS 2011, students’ achievement in reading was assessed by a performance tests that comprised items of different reading purposes (i.e., *literary* vs. *informational*) and processes of reading comprehension (e.g., *interpret and integrate ideas and information*; Mullis et al., 2009a). Following the design of TIMSS 2011, the 135 reading items were distributed among booklets in PIRLS and used for creating a set of five plausible values. Together with the plausible values obtained from TIMSS 2011, these values provide indicators of students’ achievement in mathematics, science, and reading (Martin and Mullis, 2012). For more details on the plausible value technique, as applied in TIMSS and PIRLS 2011, please refer to Foy et al. (2013).

All analyses involving the achievement scores in the three subjects are conducted with each of the five plausible values of students’ overall achievement in mathematics, science, and reading separately and the resulting model parameters (e.g., regression coefficients) are combined using the TYPE = IMPUTATION option in M*plus* (von Davier et al., 2009; Enders, 2010). The international mean of the achievement scores was set to 500 with a standard deviation of 10. In the current study, we transformed these scores by dividing them by 100 to avoid estimation problems.

### Data Analysis

#### Analytical Approach

As outlined previously, student assessments of teaching quality comprise a number of different characteristics: (i) they refer to the classroom or teacher but may also vary across individuals; (ii) they may differ across the aspects of teaching quality and subject domains; and (iii) they may differ across countries. One analytical approach that is in principle capable of accounting for these characteristics is multilevel structural equation modeling (ML-SEM; see, for example, Hox, 2013). ML-SEM is a fairly straightforward extension of ordinary SEM. The fundamental difference is that the total covariance matrix is decomposed into a between-group matrix, which includes covariances computed from group means (e.g., means for different classrooms), and a within-group matrix which includes the pooled covariances for all individuals, computed from deviations between the individuals’ scores and their respective group means (Rabe-Hesketh et al., 2007). Two different SEMs are then fitted to these two matrices in such a way that the total covariance matrix is reproduced (Cheung and Au, 2005). Thus, this modeling approach allows quite different models to account for the variation between groups and within groups. However, even though ML-SEM is straightforward in principle, it is technically a fairly demanding process to estimate such models for larger sets of data, and it has only recently started to grow in popularity (Lüdtke et al., 2009; Marsh et al., 2009; Morin et al., 2014).

#### Step 1: Establishing a Single-level Measurement Model

In order to establish a ML-SEM, we first need a measurement model for the nine items from the student questionnaire, which manages to take into account the aspects of teaching quality along with the different subject domains. One possible approach is to fit a so-called “multitrait–multimethod” confirmatory factor analysis (CFA) model, in which each observed variable typically is influenced by one “method” factor and one “trait” factor (Eid et al., 2008; Geiser et al., 2014). In our case, there would three “method” factors, each of which represents one subject (i.e., mathematics, science, and reading); and there would be three “trait” factors representing the aspects of teaching quality (i.e., teachers’ expectations, easiness to understand, and their interest in what the students say). The factors can be taken to be correlated in a so-called “oblique” model. However, in this study, another approach will be adopted by introducing a general factor which is related to all the items (see **Figure 1**, student level). This general factor accounts for the correlations among the other latent variables, transforming them into residual factors (Gustafsson and Balke, 1993; Gustafsson and Åberg-Bengtsson, 2010). Given that there are no correlations among the latent variables in the resulting model, it represents a BFSEM (Reise, 2012).

In the BFSEM, the general factor will capture individual differences in a general attitude toward the teacher and the teaching, and it will also capture general differences between countries in terms of tendency of responding positively or negatively to all the items. To the extent that such factors represent threats to the interpretability of the item responses, the general factor may be a tool for controlling for response bias due to individual response tendencies, and to cultural and language factors (He and van de Vijver, 2013). The residual subject domain and teacher aspect factors express variation with the general factor kept constant. They are, thus, to be interpreted as representing relative rather than absolute degrees of endorsement of the statements. As a first step, we specify a BFSEM for the student-level data using the TYPE = COMPLEX option in M*plus* to adjust standard errors and the chi-square statistic to the clustering of the student data in classrooms.

#### Step 2: Establishing a Multilevel Measurement Model

As has already been pointed out, we must take into account the fact that students (within level) are clustered within classrooms (between level), and that both students and classroom are systematic sources of variance in the responses. We, therefore, extend the BFSEM to a ML-BFSEM and fit one bifactor factor model to the classroom level, and one bifactor factor model to the student level, even though quite different models may be fitted to the two levels (Cheung and Au, 2005; Marsh et al., 2011). The possibility of dividing the variation into sources due to within- and between-group differences also represents a major methodological advantage, given that these sources of variation are differentially related to variation in other variables.

On the basis of these considerations, we fitted a ML-BFSEM to the pooled data from Finland, Norway, and Sweden. The hypothesized model included seven student-level factors and seven classroom-level factors (**Figure 1**). At each level, there was one general factor (*Gen*), three subject domain factors (*Math, Science*, and *Read*), and three teaching quality factors (*Expect, EasyUnd*, and *Inter*). All factors were taken to be uncorrelated with one another, and equality constraints of factor loadings were imposed for the classroom-level factors. We estimated the specificities for the general factor and subject domain factors, and the consistencies for teaching aspect factors (Eid et al., 2008).

#### Step 3: Testing for Measurement Invariance Across Countries

In order to examine whether the proposed measurement model (step 2) can be established not only for the total sample but also for each of the three countries, measurement invariance was tested (Millsap, 2011). We therefore extended the ML-BFSEM to a multi-group model and introduced equality constraints on factor loadings and intercepts in a stepwise procedure. Specifically, after specifying a model of configural invariance, which assumed the same factor structure across countries (Model MG1), we first constrained the within-level factor loadings to be equal across countries (Model MG2), and the between-level factor loadings in a second step (Model MG3). Furthermore, we examined a model with constraints on both, the within- and between-level factor loadings (full metric invariance; Model MG4), and added constraints on the item intercepts in a fourth step (Model MG5). This procedure was originally proposed by Muthén et al. (1997) and allows for a systematic investigation of multilevel latent variable modeling in multiple populations.

We evaluated the invariance models on the basis of their goodness-of-fit and the results from the model comparisons. Nevertheless, given the dependence of the χ^2^ statistic on the sample size and in light of its sensitivity toward even trivial misfit (Little, 2013), we did not rely on χ^2^ difference testing for interpreting the fit of nested models. Instead, we followed the recommendations given by Cheung and Rensvold (2002) and considered the changes of the incremental fit indices as practically insignificant if the comparative fit index (CFI) and Tucker Lewis index (TLI) changed less than 0.010, and the root mean square error of approximation (RMSEA) and standardized root mean square residual (SRMR) changed less than 0.015, compared to the configural invariance model. These statistics are particularly sensitive to deviations from invariance of factor loadings and intercepts (Chen, 2007).

#### Step 4: Estimating the Relations To Student Achievement

Under the premise that an appropriate two-level model of student assessments can be specified, such a model can then be extended by introducing further variables. We introduced students’ achievement scores in reading, mathematics, and science as correlates of the aspects of teaching quality, the subject domain factors, and the general factor.

#### Evaluation of Goodness-of-fit

In order to evaluate the model fit of the multilevel modeling approach, we refer to common guidelines (i.e., CFI ≥ 0.95, TLI ≥ 0.95, RMSEA ≤ 0.08, and SRMR ≤ 0.10 for an acceptable model fit; Marsh et al., 2005). Nevertheless, the evaluation of these goodness-of-fit indices can become quite problematic in multilevel settings, because overall fit statistics are often not sensitive enough to detect model misspecifications at the different levels of analysis (Little, 2013). Moreover, small sample sizes at the between level may not provide trustworthy fit statistics. We consequently apply the partial saturation approach in order to identify potential model misspecifications (Ryu and West, 2009; Ryu, 2014). In this approach, the factor structure of a construct is specified at one level; the other level is saturated by allowing for only correlations among the manifest indicators at the same time. This strategy backs up the simultaneous evaluation of goodness-of-fit at different levels in ML-SEM.

#### Model Estimation and Missing Data

The models are estimated with M*plus 7.3* (Muthén and Muthén, 1998–2014), using the robust maximum likelihood (MLR) estimator with standard errors, and tests of fit that are robust against non-normality of observations and the use of categorical variables in the presence of at least four response categories (Beauducel and Herzberg, 2006; Rhemtulla et al., 2012). The χ^2^ values for the models specified are corrected using the formula by Satorra and Bentler (2010). For the single-level models, the TYPE = COMPLEX option is used. The limited amount of item non-responses (less than 3.3%) is accounted for by the model-based missing-data estimation algorithm implemented in M*plus*, which yields unbiased estimates under the assumption that data is “missing at random” (i.e., full-information maximum-likelihood procedure; Enders, 2010). This assumption implies that the missing data mechanism is random, given the information in the data. To account for effects of sampling design, we use student weights (HOUWGT) in all analyses (Asparouhov, 2005). Sample M*plus* codes for the single- and ML-BFSEMs are provided in the Supplementary Material.

## Results

### Descriptive Statistics and Single-level Measurement Model

**Table 1** presents the descriptive statistics, intraclass correlations (ICC), and reliabilities for the nine items across the three countries. It may be noted that the means were considerably lower for Finland than for the other two countries, and that there was also a tendency for the Norwegian means to be higher than the Swedish means in responses. However, the sizes of the differences varied across items. The ICC-1 were around 0.10 for all the variables for the total sample, expressing that the proportion of variance due to the clustering of students in classrooms was considerable such that accounting for the clustering of students within classrooms was indicated (Hox, 2013). The ICC for the achievement measures were substantial for the total sample and the country samples (**Table 1**). Moreover, both the questionnaire items on students’ assessment of teaching quality and the achievement tests showed sufficient scale reliabilities (Cronbach’s α for *Reading*: Finland 0.85, Norway 0.86, and Sweden 0.87; *Mathematics*: Finland 0.82, Norway 0.80, and Sweden 0.80; and *Science*: Finland 0.74, Norway 0.70, and Sweden 0.77; as reported by Foy et al., 2012).

**Table 1:** Descriptive statistics, intraclass correlations (ICC), and reliabilities.

Items	Finland		Norway		Sweden		Total sample	
	*M* (*SD*)	ICC-1	*M* (*SD*)	ICC-1	*M* (*SD*)	ICC-1	*M* (*SD*)	ICC-1
**Student assessments**					
Math-Expect	3.11 (0.83)	0.034	3.55 (0.70)	0.061	3.19 (0.80)	0.044	3.28 (0.80)	0.090
Math-EasyUnd	3.38 (0.80)	0.089	3.61 (0.69)	0.050	3.56 (0.65)	0.081	3.52 (0.72)	0.092
Math-Inter	3.04 (0.88)	0.071	3.41 (0.77)	0.079	3.35 (0.75)	0.105	3.28 (0.82)	0.121
Science-Expect	3.06 (0.85)	0.035	3.52 (0.73)	0.056	3.10 (0.82)	0.061	3.21 (0.83)	0.097
Science-EasyUnd	3.36 (0.81)	0.094	3.65 (0.66)	0.052	3.53 (0.67)	0.058	3.51 (0.73)	0.098
Science-Inter	3.08 (0.90)	0.076	3.50 (0.77)	0.078	3.41 (0.75)	0.077	3.33 (0.82)	0.119
Read-Expect	3.02 (0.89)	0.033	3.44 (0.80)	0.067	3.35 (0.75)	0.046	3.27 (0.83)	0.098
Read-EasyUnd	3.41 (0.76)	0.103	3.60 (0.68)	0.045	3.56 (0.65)	0.089	3.53 (0.70)	0.096
Read-Inter	3.14 (0.83)	0.062	3.31 (0.78)	0.075	3.32 (0.73)	0.086	3.28 (0.78)	0.085
McDonald’s ω	0.87		0.83		0.82		0.86	
Cronbach’s α	0.88		0.83		0.82		0.86	
**Achievement tests**					
Mathematics	5.46 (0.64)	0.164	4.95 (0.68)	0.166	5.05 (0.67)	0.196	5.18 (0.71)	0.258
Science	5.71 (0.66)	0.150	4.94 (0.63)	0.138	5.34 (0.74)	0.250	5.38 (0.75)	0.315
Reading	5.68 (0.64)	0.158	5.07 (0.61)	0.131	5.42 (0.42)	0.214	5.43 (0.68)	0.263
Number of classrooms	267		197		251		715	
Average number of students per classroom	17.0		15.3		17.7		16.8	

*Descriptive statistics and reliabilities are reported for the student level.*

In our first step, we specified a single-level measurement model for the questionnaire items that contained a general factor (*Gen*), three factors representing the three aspects of teaching quality (*Expect, EasyUnd*, and *Inter*), and three factors representing the subject domains (*Math, Science*, and *Read*), as shown in **Figure 1** (student level). This model fitted the data excellently, Satorra-Bentler corrected (SB)-χ^2^ [9] = 26.4, *p* = 0.002, RMSEA = 0.013, CFI = 0.999, TLI = 0.997, SRMR = 0.007, and revealed significant factor loadings of the general factor (standardized λ = 0.45 – 0.68), the teaching aspect factors (*Expect*: standardized λ = 0.40 – 0.69, *EasyUnd*: standardized λ = 0.46 – 0.61, and *Inter*: standardized λ = 0.43 – 0.48), and the subject domain factors (*Math*: standardized λ = 0.12 – 0.27, *Read*: standardized λ = 0.22 – 0.24, and *Science*: standardized λ = 0.25 – 0.38). As a consequence, the specificities of teaching aspects and subjects are indicated. Please find the M*plus* code for the single-level measurement model in the Supplementary Material. Given that this single-level BFSEM provided an excellent fit and represented our hypotheses on the structure of student assessments well, it formed the baseline for further multilevel modeling.

### Multilevel BFSEM (Research Question 1)

In a second step, we extended this student-level model (within) to the classroom level (between; **Figure 1**). Since the proposed model with freely estimated factor loadings for all latent variables and at all levels did not converge due to identification problems, we constrained the unstandardized between-level factor loadings of the subject domain factors to 1, following Pohl and Steyer’s (2010) suggestions on the modeling of common trait and specific method effects. Besides these mere statistical considerations on imposing constraints to the ML-BFSEM, we had a substantive reason: Some research on the domain specificity of teaching quality suggests that the relations among different kinds of student assessments are comparable across subjects (Wagner et al., 2013). As a consequence, we decided to represent this finding in the constraints on the factor loadings of the subject domain factors to let them explain variance in the aggregated student assessment data to the same extent. The resulting model furthermore indicated that the between-level *Math* factor could not be identified due to zero variance at the classroom level. We, therefore, dropped this factor and obtained a measurement model with an excellent fit (see **Table 2**, Model M1). After saturating the within level allowing for only correlations between the manifest indicators, this model indicated an excellent fit to the data (see **Table 2**, Model M1s). However, we did not accept this model as the final measurement model for two reasons: First, in our further modeling approach of testing for measurement invariance across countries, this model (Model M1) could not be extended to a multi-group model due to non-convergence. Second, in our pursuit of parsimony and efficiency in establishing a measurement model of student assessments, we further imposed constraints on the factor loadings of the general between-level factor (*Gen*) without any important loss of model fit (see **Table 2**, Model M2). In fact, the differences between the models with and without these constraints on the general factor were moderate, ΔRMSEA = +0.004, ΔCFI = –0.003, ΔTLI = –0.004, ΔSRMR_within_ = 0.000, and ΔSRMR_between_ = +0.034 (Model M1 compared with M2). In Model M2, the SB-χ^2^ test of the resulting ML-BFSEM was highly significant, SB-χ^2^ [33] = 210.9, *p* < 0.001. However, the data set is very large, which leads to a high power for detecting even trivial deviations from the model; hence, the significant χ^2^ test does not necessarily indicate serious misfit. According to further descriptive fit indices, the model fitted the data excellently (see **Table 2**, Model M2).

**Table 2:** Fit statistics of the ML-BFSEM with different constraints (total sample).

Model	Constraints within the model	SB-χ ^2^ [*df*]	RMSEA	CFI	TLI	SRMR_within_	SRMR_between_
**Total Sample**					
M1	Equal loadings of the between-level subject domain factors	115.9 [25]^∗^	0.017	0.997	0.992	0.005	0.036
M1s	See M1 + saturated within level	99.0 [16]^∗^	0.021	0.997	0.988	0.001	0.026
M2	Equal loadings of the between-level subject domain factors + general factor	210.9 [33]^∗^	0.021	0.994	0.988	0.005	0.070
M2s	See M2 + saturated within level	200.5 [24]^∗^	0.025	0.994	0.983	0.002	0.070
**Finland**					
M2	Equal loadings of the between-level subject domain factors + general factor	78.7 [33]^∗^	0.017	0.997	0.994	0.005	0.079
M2s	See M2 + saturated within level	77.2 [24]^∗^	0.022	0.997	0.990	0.003	0.079
**Norway**					
M2	Equal loadings of the between-level subject domain factors + general factor	94.4 [33]^∗^	0.025	0.992	0.983	0.012	0.095
M2s	See M2 + saturated within level	78.8 [24]^∗^	0.027	0.993	0.979	0.002	0.095
**Sweden**					
M2	Equal loadings of the between-level subject domain factors + general factor	89.3 [34^a^]^∗^	0.019	0.994	0.988	0.006	0.103
M2s	See M2 + saturated within level	82.1 [24]^∗^	0.023	0.994	0.982	0.002	0.102

*SB-χ^*2*^ [*df*] = Satorra-Bentler corrected χ^*2*^ statistic with *df* degrees of freedom.*

*^a^In order to estimate this model, the residual variance of one item had to be constrained to zero; ^∗^*p* < 0.001.*

**FIGURE 2:**
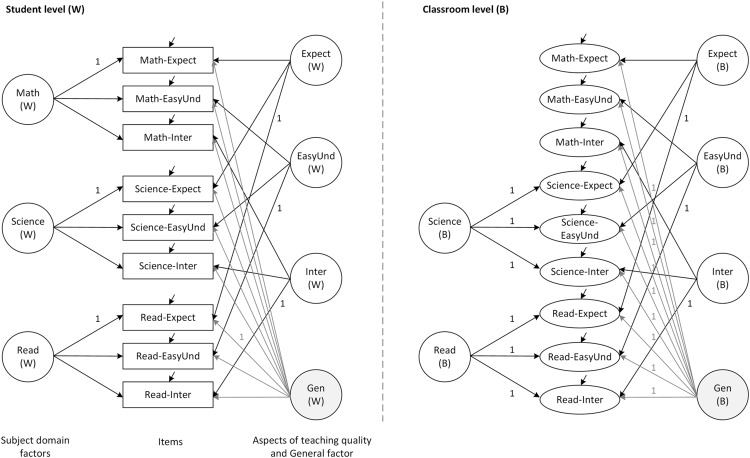
**Final multilevel bifactor structural equation model of student assessments.** Math, Mathematics; Read, Reading; Expect, Teacher expectations; EasyUnd, Easy to understand; Inter, Interest; Gen, General factor.

In order to test for potential misspecifications at the student- or classroom-level and to support the findings on the structure of student assessments, we followed the partial saturation approach (Ryu and West, 2009; Ryu, 2014). Specifically, we saturated the student level and specified the measurement model at the classroom level, yielding an acceptable fit (see **Table 2**, Model M2s). These findings support the good fit of the overall ML-BFSEM to the data of the total sample. In sum, the empirical model of students’ assessments of teaching quality comprised three teacher aspect factors, a general factor, and two subject domain factors (for reading and science) at the between level, and contained constraints on the factor loadings of the general and subject domain factors, as shown in **Figure 2**. Please find the M*plu*s sample code of this model in the Supplementary Material.

**Table 3** presents the standardized factor loadings for the within and the between models for the total sample. The loadings on the general factor *at the student level* were all quite substantial, varying between 0.4 and 0.6. The corresponding specificities were moderate. Additionally, the three teaching aspect factors had high relations to the indicators, most of them being in the range of 0.4–0.6 and leading to moderate to high consistencies. A tendency can also be observed for the loadings on the mathematics items to be higher than for the other domains. For the three subject domain factors, all loadings were highly significant, but the estimates were lower for these factors, typically being in the range of 0.2–0.3. Moreover, low domain specificities were indicated.

**Table 3:** Standardized factor loadings, consistencies, and specificities of the ML-BFSEM (total sample).

Items	General factor	Read	Math	Science	Expect	EasyUnd	Inter	GENFS	DOS	CON
**Student (within) level**					
Read-Expect	0.44	0.23	–	–	0.41	–	–	0.46	0.12	0.41
Read-EasyUnd	0.55	0.27	–	–	–	0.50	–	0.49	0.11	0.40
Read-Inter	0.58	0.24	–	–	–	–	0.46	0.56	0.09	0.34
Math-Expect	0.38	–	0.20	–	0.70	–	–	0.21	0.06	0.73
Math-EasyUnd	0.60	–	0.23	–	–	0.59	–	0.48	0.07	0.45
Math-Inter	0.63	–	0.20	–	–	–	0.51	0.57	0.06	0.37
Science-Expect	0.40	–	–	0.31	0.64	–	–	0.24	0.14	0.62
Science-EasyUnd	0.62	–	–	0.35	–	0.44	–	0.55	0.18	0.27
Science-Inter	0.60	–	–	0.31	–	–	0.45	0.55	0.15	0.31
**Classroom (between) level**					
Read-Expect	0.73	0.15	–	–	0.49	–	–	0.68	0.03	0.30
Read-EasyUnd	0.89	0.18	–	–	–	0.39	–	0.82	0.03	0.15
Read-Inter	0.85	0.17	–	–	–	–	0.47	0.74	0.03	0.23
Math-Expect	0.82	–	–	–	0.54	–	–	0.70	–	0.30
Math-EasyUnd	0.91	–	–	–	–	0.40	–	0.84	–	0.16
Math-Inter	0.83	–	–	–	–	–	0.46	0.77	–	0.23
Science-Expect	0.77	–	–	0.30	0.51	–	–	0.63	0.10	0.28
Science-EasyUnd	0.87	–	–	0.34	–	0.38	–	0.75	0.11	0.14
Science-Inter	0.79	–	–	0.31	–	–	0.44	0.69	0.10	0.21

*Standardized factor loadings are statistically significant at the 0.1%-level. GENFS, general factor specificity; DOS, domain (subject) specificity; CON, consistency (i.e., teaching aspects specificity).*

The loadings on the general factor *at the classroom level* were very high, and varied between 0.7 and 0.9. The dominance of this factor was indicated by the high specificities. For the reading factor, the loadings were around 0.15, yet significant. No mathematics factor could be identified at the between level, the estimated variance being just below 0. We therefore restricted the factor loadings of this factor to zero in the model. Moreover, the reading factor was weakly identified, as indicated by the low factor loadings and domain specificities. However, for the science factor, the loadings were around 0.3 and highly significant. Furthermore, all three teaching aspects had highly significant loadings in the range of 0.4–0.5 and showed substantial consistencies.

Finally, we specified the ML-BFSEM for each country sample individually in order to examine whether this model may serve as a baseline for further multi-group modeling approaches (**Figure 2**). In fact, this model fitted the data well for each of the country samples (see **Table 2**, Model M2 for each country); and even after saturating the within level, the acceptable model fit remained (see **Table 2**, Model M2s for each country).

In response to Research Question 1, we point out that the measurement model could identify all the hypothesized factors at the student level. Five of the seven hypothesized factors could be identified at the classroom level, there being little or no variance and specificity in the two of the subject domain factors (i.e., *Math* and *Read*; **Table 3**). There is reason, however, to continue the analysis of the relations between the three achievement measures and the identified factors. Overall, the ML-BFSEM approach resulted in an acceptable goodness-of-fit and could be established for both the total sample and the individual country samples. It consequently forms the baseline for further measurement invariance testing.

### Multi-group Ml-BFSEM (Research Question 2)

Our second research question was concerned with the invariance of the ML-BFSEM across the three countries. Addressing Research Question 1, we already had evidence that this model can be specified for each country sample with an acceptable model fit (**Table 2**). On the basis of this finding, we established the configural invariance model as the baseline. This model showed an acceptable fit and could, therefore, be accepted (see **Table 4**, Model MG1). As further restrictions on the within-level (Model MG2) or between-level factor loadings (Model MG3) were imposed, the resulting models still showed acceptable goodness-of-fit statistics (**Table 4**). Moreover, the model fit did not change substantially when comparing models MG1 and MG2 (ΔRMSEA = +0.005, ΔCFI = –0.006, ΔTLI = –0.007, ΔSRMR_within_ = +0.012, and ΔSRMR_between_ = +0.001), and models MG1 and MG3 (ΔRMSEA = 0.000, ΔCFI = 0.000, ΔTLI = –0.001, ΔSRMR_within_ = 0.000, and ΔSRMR_between_ = +0.005). Bringing the equality constraints on the within- and between-level factor loadings together in one model (Model MG4), we still found fit and acceptable changes in the fit statistics compared to the configural model, ΔRMSEA = +0.005, ΔCFI = –0.006, ΔTLI = –0.006, ΔSRMR_within_ = +0.012, and ΔSRMR_between_ = +0.006. As a consequence, we accepted this model and concluded that full metric invariance was met. Finally, we constrained the item intercepts which only exist at the between level (Model MG5). Again, the resulting model had an acceptable fit (**Table 4**), and the changes in the fit statistics were within the suggested boundaries, ΔRMSEA = +0.005, ΔCFI = –0.007, ΔTLI = –0.007, ΔSRMR_within_ = +0.012, and ΔSRMR_between_ = +0.003. Hence, scalar invariance could also be established.

**Table 4:** Fit statistics of the multi-group ML-BFSEM with different constraints (invariance testing).

Model	Equality constraints across groups	SB-χ ^2^ [*df*]	RMSEA	CFI	TLI	SRMR_within_	SRMR_between_
**Measurement invariance**					
MG1	Within and between-level factor structure (*configural invariance*)	264.4 [102]^∗^	0.020	0.995	0.990	0.008	0.093
MG2	Within-level factor loadings (*within-level metric invariance*)	503.5 [141]^∗^	0.025	0.989	0.983	0.020	0.094
MG3	Between-level factor loadings (*between-level metric invariance*)	291.5 [111]^∗^	0.020	0.995	0.989	0.008	0.098
MG4	Within- and between-level factor loadings (*full metric invariance*)	521.0 [151]^∗^	0.025	0.989	0.984	0.020	0.099
MG5	Within- and between-level factor loadings + item intercepts (*scalar invariance*)	561.9 [157]^∗^	0.025	0.988	0.983	0.020	0.096
**Invariance of structural parts**					
MG6	See MG5 + between-level factor variances	569.2 [170]^∗^	0.024	0.988	0.985	0.020	0.106
MG7	Freely estimated relations to achievement	767.7 [229]^∗^	0.024	0.990	0.983	0.017	0.080
MG8	See MG7 + within-level relations to achievement	844.6 [263]^∗^	0.023	0.989	0.984	0.022	0.080
MG9	See MG8 + between-level relations to achievement	895.8 [289]^∗^	0.023	0.989	0.985	0.022	0.111

*MG, Multi-group model; SB-χ^*2*^ [*df*] = Satorra–Bentler corrected χ^*2*^ statistic with *df* degrees of freedom. Models MG2 to MG9 assume the same factor structure across countries. ^∗^*p* < 0.001.*

In summary, the invariance testing suggested that both metric and scalar invariance were met for the ML-BFSEM approach. As a consequence, this approach allowed us to compare the relations to further variables such as student achievement and, in addition, the factor means across the three Nordic countries.

### Relations Between Student Assessments of Teaching Quality and Achievement (Research Question 3)

In order to investigate the relations between the factors identified within the questionnaire data and achievement at the two levels of observation (Research Question 3), the plausible values representing achievement in each of the three subject domains were regressed onto the latent variables at both the within and the between level. We examined these relations in different steps: first, we tested whether or not the relations to achievement were invariant across countries. If this was the case, we could proceed by analyzing the pooled data set without accounting for the multi-group structure in a second step. We would thereby allow for small country-specific deviations from the resulting regression parameters. If this was not the case, we could proceed with a more complex multi-group model, in which the relations to achievement were freely estimated for each country sample.

To identify potential sources of variation in the relations to student achievement, we tested whether the between-level factor variances showed invariance across the three countries. The resulting model fitted the data sufficiently (see **Table 4**, Model MG6) and indicated no substantial loss in model fit compared to the configural model, ΔRMSEA = +0.004, ΔCFI = –0.007, ΔTLI = –0.005, ΔSRMR_within_ = +0.012, and ΔSRMR_between_ = +0.013, showing that factor variance can be regarded as invariant. Subsequently, the regression part was added to the multi-group model, and the relations to achievement were freely estimated (for a sample M*plus* code, please refer to the Supplementary Material). As it was not possible to estimate this extended model with all predictors due to non-convergence, we had to exclude the factor *Inter* from the list of predictors at the between level. This model showed an acceptable fit to the data and formed the basis for testing the effects of further restrictions on the structural relations (**Table 4**, Model MG7). Specifically, constraining the within-level relations to achievement did not change the model fit substantially (ΔRMSEA = –0.001, ΔCFI = –0.001, ΔTLI = +0.001, ΔSRMR_within_ = +0.005, and ΔSRMR_between_ = 0.000). Additionally, this model had an acceptable goodness-of-fit (see **Table 4**, Model MG8). Furthermore, even restricting the between-level relations in addition to the constraints in Model MG8 resulted in an acceptable fit (see **Table 4**, Model MG9) and a marginal loss in fit, ΔRMSEA = –0.001, ΔCFI = –0.001, ΔTLI = +0.002, ΔSRMR_within_ = +0.005, and ΔSRMR_between_ = +0.031. We note that the SRMR_between_ changed after constraining the between-level relations and was slightly higher than the suggested cut-off (0.010). However, since only little is known about the performance of this fit statistic in multilevel settings (Hsu et al., 2015) and a number of studies on teaching quality found similar values of the SRMR_between_ (Wagner et al., 2013; Fauth et al., 2014), we accepted Model MG9 and argued that the relations to student achievement were invariant across countries. These analyses formed the basis for describing the relations by using the pooled data set rather than adopting a more complex multi-group approach.

In a next step, we introduced the relations to student achievement at the student and the classroom level, and two dummy-coded variables representing country membership (*Finland* and *Norway*, with Sweden taken as the reference group). In this model, students’ achievement in the three subjects was predicted by the teacher aspects and the subject domain factors at the student level. At the classroom level, achievement was predicted by the teacher aspect factors and the two dummy variables, but not by the subject domain factors. The latter choice was made, because we were more interested in the relations of student achievement to the teaching aspects than to students’ aggregated perceptions of a subject domain, following the teaching effectiveness research tradition (Creemers and Kyriakides, 2008). Since the inclusion of the factor *Inter* at the within level led to an overestimation of correlations between some of the factors, we had to exclude this latent variable from the model. Finally, the resulting model could be estimated and showed an acceptable fit, SB-χ^2^ [77] = 676.6, *p* < 0.001, RMSEA = 0.025, CFI = 0.989, TLI = 0.977, SRMR_within_ = 0.009, and SRMR_between_ = 0.068. We further note that this model contained the *Math* factor at the student level only. The relations to student achievement are shown in **Table 5**.

**Table 5:** Standardized regression coefficients describing the relations between student assessments of teaching quality and achievement in different subject domains for the pooled sample.

β (SE)	General factor	Expect	Inter	EasyUnd	Math	Science	Read
**Student (within) level**					
Mathematics	0.00 (0.02)	-0.03 (0.01)^∗^	–	0.06 (0.02)^∗∗∗^	0.18 (0.03)^∗∗∗^	–	0.12 (0.03)^∗∗∗^
Reading	0.05 (0.02)^∗∗^	0.00 (0.01)	–	0.07 (0.02)^∗∗∗^	–	–	0.13 (0.02)^∗∗∗^
Science	0.01 (0.02)	0.01 (0.02)	–	0.07 (0.02)^∗∗∗^	–	0.04 (0.03)	0.10 (0.03)^∗∗∗^
**Classroom (between) level**					
Mathematics	-0.13 (0.08)	0.26 (0.18)	–	0.31 (0.09)^∗∗∗^	–	–	–
Reading	-0.13 (0.08)	0.23 (0.17)	–	0.32 (0.09)^∗∗∗^	–	–	–
Science	-0.20 (0.07)^∗∗^	0.27 (0.16)	–	0.32 (0.08)^∗∗∗^	–	–	–

*^∗^*p* < 0.05, ^∗∗^*p* < 0.01, and ^∗∗∗^*p* < 0.001.*

#### Relations in the Within-level Model

**Table 5** presents the significant relations between the latent variables in the within-level model and the three achievement measures. For all achievement variables, there was a significant relation to *EasyUnd*. There also was a significant relation from *Read* to all achievement measures; in addition, the *Math* factor predicted achievement in mathematics. For the reading achievement measure, the *Gen* factor had a significant relation (β = 0.05, *SE* = 0.02, *p* < 0.01). Moreover, mathematics achievement was slightly negatively related to students’ perceptions of clear teacher expectations (β = –0.03, *SE* = 0.01, *p* < 0.05). This pattern of relations between the subject domain factors and the achievement variables showed that students tend to evaluate the teacher positively in the domains where they have performance strengths. This provides some validation of the analytical approach. However, it must be emphasized that the causal relation may go either from the positive assessment of the teacher to achievement, or the other way around, or in both directions. It can also be observed that the only teaching aspect that is related to achievement in all three subjects refers to students’ assessment of how easy the teacher is to understand (*EasyUnd*).

#### Relations in the Between-level Model

The results for the between level were quite simple and clear-cut, that is that only the *EasyUnd* factor had any relation to the three achievement measures (**Table 5**). In addition, the general factor was negatively related to classroom-level achievement in science (β = –0.20, *SE* = 0.07, *p* < 0.01).

#### Mediation of Country Differences in Achievement by Student Assessments

In addition to the positive relation between the *EasyUnd* factor and achievement in the between-level model, there also were substantial differences in the means of this factor across countries. The mean for Finland was 1.05 standard deviation units (*d*; *t* = 6.38, *p* < 0.001) higher than the mean of *EasyUnd* for Sweden, while there was no significant difference between Norway and Sweden (*d* = 0.32, *t* = 1.86, *p* = 0.06). This pattern of results suggested that the country differences in the levels of achievement could at least partially be mediated by the country differences in students’ assessment of how well they understand their teachers.

**FIGURE 3:**
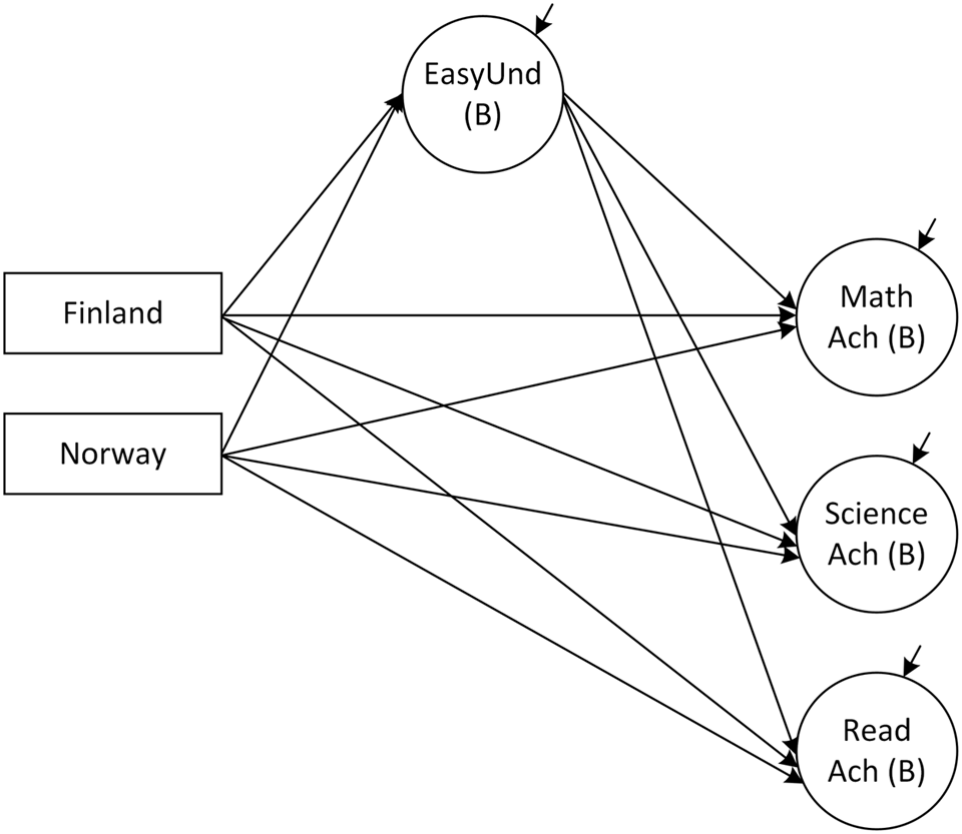
**Mediation part of the model describing the relations to student achievement at the classroom level (B).** *Ach*, Achievement; *EasyUnd*, Easy to understand. *Finland* and *Norway* represent dummy-coded variables of country membership.

Testing the direct and indirect effects in the mediation model, which considers *EasyUnd* to be a mediator variable between country membership and achievement (**Figure 3**), revealed significant indirect effects for *Finland* but not for *Norway* (**Table 6**). In addition, the direct effects of country membership on mathematics and science achievement remained significant for both dummy variables. Hence, at least partial mediation of the relation between *Finland* and achievement in mathematics and science can be assumed. Only for reading achievement, a full mediation was detected, as indicated by the insignificant direct effect (β = 0.06, *SE* = 0.15, *p* > 0.05) and the significant indirect effect (β = 0.12, *SE* = 0.04, *p* < 0.01). The main conclusion therefore is that the differences in the levels of achievement between Finland on the one hand and Sweden and Norway on the other hand can be accounted for by the student-assessed differences in how well they understand the teacher. Since there was only a small difference between Swedish and Norwegian students in this teaching aspect, none of the differences in the levels of achievement between the two countries was mediated by *EasyUnd*. These analyses demonstrate how flexibly the ML-BFSEM can be used to study cross-country differences.

**Table 6:** Direct and indirect effects of dummy-coded country variables on achievement via the factor EasyUnd at the classroom level (B).

β (SE)	Mathematics achievement	Science achievement	Reading achievement
Dummy variable: Norway			
Direct effect	-0.09 (0.04)^∗^	-0.37 (0.04)^∗∗∗^	0.33 (3.63)
Indirect effect via *EasyUnd (B)*	0.03 (0.02)	0.03 (0.02)	0.03 (0.02)
Dummy variable: Finland			
Direct effect	0.47 (0.06)^∗∗∗^	0.35 (0.05)^∗∗∗^	0.06 (0.15)
Indirect effect via *EasyUnd (B)*	0.11 (0.04)^∗∗^	0.10 (0.04)^∗∗^	0.12 (0.04)^∗∗^

*The table shows the fully standardized results. ^∗^*p* < 0.05, ^∗∗^*p* < 0.01, and ^∗∗∗^*p* < 0.001.*

## Discussion

The main objective of the present study was to illustrate the application of a multilevel bifactor structural equation modeling approach in describing the structure of student assessments of teaching quality and their relations to achievement in three Nordic countries. Our secondary objectives referred to increasing the popularity of ML-BFSEM and creating an awareness of its usefulness in modeling student assessments of teaching quality. Approaching these objectives, we found that a ML-BFSEM fitted the data very well and represented the theoretical assumptions on the structure of teaching quality. Specifically, this model assumed three factors representing the aspects of teaching quality, three subject domain factors representing the domain-specificity of teaching quality, and a general factor at each level of analysis representing general response tendencies or perceptions of teaching quality. We note that alternative modeling approaches of the multilevel CFA tradition mostly exclude one of these assumptions. In fact, a number of studies exist which take into account the multilevel and multidimensional structure of teaching quality, but do not specify subject domain factors or a general factor (Wagner et al., 2013; Fauth et al., 2014). From a substantive point of view, the ML-BFSEM uniquely represents an integrative approach to describe teaching quality. However, we encourage further methodological research to disentangle how well the ML-BFSEM performs in comparison with alternative models in simulations studies. Nevertheless, our study has shown that the ML-BFSEM approach is suitable in terms of model fit and describing the relations to other constructs.

In the ML-BFSEM, the teaching quality factors and the general factor showed high specificities, whereas the subject domain factors were weakly identified yet existent at the classroom level. The between-level relations to achievement were significant only for the factor *EasyUnd*; and country differences in achievement were partially mediated by this factor. The student-level model also showed a meaningful pattern of relations between achievement in the three subjects and student evaluation of teaching in these areas, and particularly so for mathematics and science.

One of the key findings in our study was the persistence of the three teaching quality factors. Both at the student and the classroom level, these factors could be identified with high factor loadings and specificities. This finding is in line with existing research on the structure of teaching quality assessments, which adopted multilevel CFA (Wagner et al., 2013; Fauth et al., 2014). It seems as if students are generally able to distinguish between different aspects of teaching. Hence, this can be interpreted as evidence for the internal validity of student assessments (Kunter and Baumert, 2006). We note that, in contrast to other studies, we assumed uncorrelated teaching quality factors and captured their relations by a general factor. This approach seems advantageous for at least two reasons: first, it assumes that a general factor underlies students’ responses in all items, which reflects a robust assumption for most psychological constructs (Gustafsson and Åberg-Bengtsson, 2010; Aguado et al., 2015; Morin et al., 2015). In fact, classroom perceptions and the “psychological climate” are often influenced by a general trait or response tendency (Parker et al., 2003; He and van de Vijver, 2013). Second, given that the factors are uncorrelated in the overall model, the relations to external variables such as achievement can be described without potential biases that are due to multicollinearity of the predictors (Reise, 2012). Moreover, the correlations between some of the teaching quality aspects have been reported to be high at the classroom level, sometimes exceeding 0.70 (Wagner et al., 2013). As a consequence, we believe that the assumption of a general factor is reasonable (see also Morin et al., 2014).

In our pursuit of making the case for the ML-BFSEM approach, we tested for measurement invariance across countries. Interestingly, both metric and scalar invariance could be established, enabling us to compare relations to other variables and factor means. Given these degrees of comparability of the measurement model, we see evidence for the persistence of the hypothesized factor structure of student assessments (Millsap, 2011). As a consequence, we consider this finding to be another element in support of internal validity.

It is quite worthwhile mentioning that the general between-level factor did not have any relation to the achievement measures expect for science, and any tendency that could be observed was negative rather than positive. This strengthens the interpretation that this factor does not carry any substantive meaning, but that it rather is a method factor caused by the students in different classrooms and countries using the response scale in a more or less positive manner, without this being related to the quality of the different aspects being evaluated. It is, indeed, quite a difficult task for fourth-graders to evaluate the quite abstract aspects of teaching on a four-point scale, given that many of them had experienced only one or a few teachers during their life in school so far (Fauth et al., 2014). This also makes the evaluations sensitive to any stereotypical tendency to more or less positive responses (He and van de Vijver, 2013).

It is also interesting to note that the only classroom-level factor that had any relation to achievement in all subject domains was the evaluation of the degree to which the teacher was easy to understand. One reason for this may be that this teaching aspect may be comparatively easy for the students to assess, and particularly so since they could do this in the context of the two other aspects. It may thus be hypothesized that the students assessed the quality of teaching by making relative comparisons between the three aspects of quality. Another reason may, of course, be that this aspect is the only factor which has a relation to student achievement, either because there is a causal relation from the students’ understanding of the teaching to student achievement, or because students who achieve well for some other reason will also experience that they understand the teacher (Titsworth et al., 2015). It should be emphasized that at the classroom-level too it is necessary to be cautious when interpreting this relation in causal terms, even though it does seem more reasonable at this level than at the student-level to interpret the relation as being due to an effect of teaching on achievement.

However, even though these relations seem reasonable, it could also be argued that some expected relations have not been established. For instance, the factor representing teacher’s expectation on achievement provides an example, where the classroom-level factor showed considerable variation, and differences between countries, but which was not related to achievement differences at the classroom level. One possible explanation for the lack of expected findings may be that the information in the nine items is not sufficient to estimate with precision all of the 14 hypothesized latent variables in the ML-BFSEM. One way to test this hypothesis is to continue the kind of analyses presented here, using other and richer sources of data, which contain a larger number of items that measure further aspects of teaching quality (Wagner et al., 2013).

Another challenge is that the ML-BFSEM and its extensions represent multilevel latent variable models with a large number of model parameters (Van De Schoot et al., 2015). The complexity of these models may, therefore, require imposing constraints or “sacrificing” factors that are weakly identified. In the current study, we decided to constrain some of the factor loadings at the between level, and dropped one of the three subject domain factors (*Math*). Although such constraints reduce the information that could potentially be provided on, for instance, the relations to other variables such as student achievement, the remaining findings were still robust, for instance, with respect to the factor structure identified across countries. Nevertheless, we encourage further methodological research to compare the convergence, performance, and specification of the ML-BFSEM to alternative modeling approaches.

An interesting challenge in analyzing these data is that each particular item combines two different facets, namely the subject domain and the aspects of teaching quality. In order to understand the influence of the different aspects of teaching on the one hand, and the differences between the three subjects on the other hand, it is necessary to disentangle the two facets. In this regard, we would like to point out that, although our ML-BFSEM approach identified three subject domain factors at the student level and only one factor at the classroom level, the effects of domain specificity were very low. This finding may suggest the generalizability of student assessments of teaching quality across subjects (Wagner et al., 2013). Nevertheless, this may also be a result of the specific aspects of teaching quality. Klieme et al. (2009) argued that domain specificity manifests in aspects of teaching quality, which are closely related to the subject-specific teaching strategies such as cognitive activation. In contrast, the effects may be lower for generic teaching aspects, which were assessed in TIMSS and PIRLS 2011.

It is quite obvious that it had not been possible to identify the relations between students’ evaluation of the teacher/teaching and student achievement unless a latent variable modeling approach had been adopted. One reason for this is that the item responses are complex, each item being influenced by both a subject domain and a teaching aspect, along with item-specific information and random variation. Unless the item information is reorganized in such a way that these sources of variation are captured in different dimensions, it will be impossible to determine their relations to other variables (Eid et al., 2008; Geiser et al., 2015). Furthermore, an analytical approach is required which can separate variation between classrooms and between students within classrooms. In the data, the former source of variation only accounts for some 10% of the total variation in each item, and given that this information is of central interest when trying to explain impact of teachers and teaching on achievement, it is necessary to explicitly separate it from the variation due to students.

It is also obvious, however, that the validity of the inferences depends on the quality of information analyzed, and on how reasonable the model and its assumptions are for the phenomenon at hand (Messick, 1995). It does seem that the results from the student-level model do make sense, even though it must be emphasized that the relations between the student evaluations of teaching quality should not be interpreted as causing student achievement. It is just as reasonable to expect that a high level of student achievement causes the students to evaluate the teacher positively (Klieme, 2013).

## Conclusion

The ML-BFSEM approach adopted in the current study allows researchers to account for the different aspect of teaching quality, subject domains, and potential cross-cultural response biases in a straightforward way. It furthermore provides reasonable evidence for construct validity with respect to the internal structure and the relations to external variables. We encourage the application of the ML-BFSEM in the field of educational effectiveness.

## Conflict of Interest Statement

The authors declare that the research was conducted in the absence of any commercial or financial relationships that could be construed as a potential conflict of interest.
