# Supplementary material for: Student assessment of teaching as a source of information about aspects of teaching quality in multiple subject domains: an application of multilevel bifactor structural equation modeling
Source: Front Psychol. 2015 Oct 8;6:1550. doi: 10.3389/fpsyg.2015.01550 (PMC4597036; doi:10.3389/fpsyg.2015.01550)
Supplement: Supplementary file 1 [file Table_1.DOCX]

# *Supplementary Material*

**Student Assessment of Teaching as a Source of Information about Aspects of Teaching Quality in Multiple Subject Domains: An Application of Multilevel Bifactor Structural Equation Modeling**

**Ronny Scherer*, Jan-Eric Gustafsson**

*** Correspondence:** Ronny Scherer: ronny.scherer@cemo.uio.no

**M*plus* sample code of the single- and multilevel bifactor structural equation models**

1. Single-level bifactor structural equation model of student assessments

TITLE: Single-level BFSEM of student assessments

DATA: FILE IS timss2011.dat;

VARIABLE: NAMES ARE IDCLASS IDSTUD HOUWGT FIN NOR RDEXP RDEASY RDINT MAEXP MAEASY MAINT SCEXP SCEASY SCINT;

! Item labeling:

! RD = Reading, MAT = Mathematics, SC = Science

! EXP = Expect, EASY = EasyUnd, INT = Inter

USEVARIABLES ARE RDEXP-SCINT;

CLUSTER = IDCLASS;

! Classroom ID as the cluster variable

WEIGHT = HOUWGT;

! House weights to account for sampling bias

MISSING ARE ALL(-99);

! Missing values are specified as -99

ANALYSIS: TYPE = COMPLEX;

ESTIMATOR = MLR;

H1ITERATIONS = 4000;

MODEL: ! Specify the general factor Gf

Gf BY

RDEXP@1

RDEASY*

RDINT*

MAEXP*

MAEASY*

MAINT*

SCEXP*

SCEASY*

SCINT*;

! Specify the teaching aspect factors

Expect BY

RDEXP@1

MAEXP*

SCEXP*;

EasyUnd BY

RDEASY@1

MAEASY*

SCEASY*;

Inter BY

RDINT@1

MAINT*

SCINT*;

! Specify the subject domain factors

Read BY

RDEXP@1

RDEASY*

RDINT*;

Math BY

MAEXP@1

MAEASY*

MAINT*;

Science BY

SCEXP@1

SCEASY*

SCINT*;

! Freely estimate factor and residual variances

Gf-Science*;

RDEXP-SCINT*;

! Restrict factor covariances to zero

Gf-Science WITH Gf-Science@0;

! Freely estimate item intercepts

[RDEXP-SCINT*];

OUTPUT: stdyx;

1. Multilevel bifactor structural equation model of student assessments (measurement model)

TITLE: ML-BFSEM of student assessments

DATA: FILE IS timss2011.dat;

VARIABLE: NAMES ARE IDCLASS IDSTUD HOUWGT FIN NOR RDEXP RDEASY RDINT MAEXP MAEASY MAINT SCEXP SCEASY SCINT;

! Item labelling:

! RD = Reading, MAT = Mathematics, SC = Science

! EXP = Expect, EASY = EasyUnd, INT = Inter

USEVARIABLES ARE RDEXP-SCINT;

CLUSTER = IDCLASS;

! Classroom ID as the cluster variable

WEIGHT = HOUWGT;

! House weights to account for sampling bias

MISSING ARE ALL(-99);

! Missing values are specified as -99

ANALYSIS: TYPE = TWOLEVEL;

ESTIMATOR = MLR;

H1ITERATIONS = 4000;

MODEL: %WITHIN%

! Student level (W)

! Specify the general factor GfW

GfW BY

RDEXP@1

RDEASY*

RDINT*

MAEXP*

MAEASY*

MAINT*

SCEXP*

SCEASY*

SCINT*;

! Specify the teaching aspect factors (W)

ExpectW BY

RDEXP@1

MAEXP*

SCEXP*;

EasyUndW BY

RDEASY@1

MAEASY*

SCEASY*;

InterW BY

RDINT@1

MAINT*

SCINT*;

! Specify the subject domain factors (W)

ReadW BY

RDEXP@1

RDEASY*

RDINT*;

MathW BY

MAEXP@1

MAEASY*

MAINT*;

ScienceW BY

SCEXP@1

SCEASY*

SCINT*;

! Freely estimate factor and residual variances

GfW-ScienceW*;

RDEXP-SCINT*;

! Restrict factor covariances to zero

GfW-ScienceW WITH GfW-ScienceW@0;

%BETWEEN%

! Classroom level (B)

! Specify the general factor GfB

GfB BY

RDEXP@1

RDEASY@1

RDINT@1

MAEXP@1

MAEASY@1

MAINT@1

SCEXP@1

SCEASY@1

SCINT@1;

! Specify the teaching aspect factors (B)

ExpectB BY

RDEXP@1

MAEXP*

SCEXP*;

EasyUndB BY

RDEASY@1

MAEASY*

SCEASY*;

InterB BY

RDINT@1

MAINT*

SCINT*;

! Specify the subject domain factors (B)

ReadB BY

RDEXP@1

RDEASY@1

RDINT@1;

ScienceB BY

SCEXP@1

SCEASY@1

SCINT@1;

! Freely estimate factor and residual variances

GfB-ScienceB*;

RDEXP-SCINT*;

! Restrict factor covariances to zero

GfB-ScienceB WITH GfB-ScienceB@0;

! Freely estimate item intercepts

[RDEXP-SCINT*];

OUTPUT: stdyx;

1. Multi-group multilevel bifactor structural equation model of student assessments including the relations to student achievement

TITLE: Multi-group ML-BFSEM of student assessments and freely estimated relations to student achievement

DATA: FILE IS timss2011_list.dat;

TYPE = IMPUTATION;

! Run the analyses for each of the five plausible

! values and combine the results (model parameter)

VARIABLE: NAMES ARE IDCLASS IDSTUD HOUWGT FIN NOR RDEXP RDEASY RDINT MAEXP MAEASY MAINT SCEXP SCEASY SCINT

RDACH MATACH SCACH CNT;

! Item labelling:

! RD = Reading, MAT = Mathematics, SC = Science

! EXP = Expect, EASY = EasyUnd, INT = Inter

! ACH = Achievement

USEVARIABLES ARE RDEXP-SCACH;

CLUSTER = IDCLASS;

! Classroom ID as the cluster variable

WEIGHT = HOUWGT;

! House weights to account for sampling bias

MISSING ARE ALL(-99);

! Missing values are specified as -99

GROUPING IS CNT (1=FIN 2=NOR 3=SWE);

! Specification of the grouping variable (country)

ANALYSIS: TYPE = TWOLEVEL;

ESTIMATOR = MLR;

H1ITERATIONS = 4000;

MODEL: %WITHIN%

! Student level (W)

! Specify the general factor GfW

GfW BY

RDEXP@1

RDEASY*

RDINT*

MAEXP*

MAEASY*

MAINT*

SCEXP*

SCEASY*

SCINT*;

! Specify the teaching aspect factors (W)

ExpectW BY

RDEXP@1

MAEXP*

SCEXP*;

EasyUndW BY

RDEASY@1

MAEASY*

SCEASY*;

InterW BY

RDINT@1

MAINT*

SCINT*;

! Specify the subject domain factors (W)

ReadW BY

RDEXP@1

RDEASY*

RDINT*;

MathW BY

MAEXP@1

MAEASY*

MAINT*;

ScienceW BY

SCEXP@1

SCEASY*

SCINT*;

! Freely estimate factor and residual variances

GfW-ScienceW*;

RDEXP-SCINT*;

! Restrict factor covariances to zero

GfW-ScienceW WITH GfW-ScienceW@0;

! Relations to student achievement

! Teacher aspects and general factor as predictors

RDACH MATACH SCACH ON GfW-InterW;

! Subject domain factors as predictors

RDACH ON ReadW;

MATACH ON MathW ReadW;

SCACH ON ScienceW ReadW;

%BETWEEN%

! Classroom level (B)

! Specify the general factor GfB

GfB BY

RDEXP@1

RDEASY@1

RDINT@1

MAEXP@1

MAEASY@1

MAINT@1

SCEXP@1

SCEASY@1

SCINT@1;

! Specify the teaching aspect factors (B)

ExpectB BY

RDEXP@1

MAEXP*

SCEXP*;

EasyUndB BY

RDEASY@1

MAEASY*

SCEASY*;

InterB BY

RDINT@1

MAINT*

SCINT*;

! Specify the subject domain factors (B)

ReadB BY

RDEXP@1

RDEASY@1

RDINT@1;

ScienceB BY

SCEXP@1

SCEASY@1

SCINT@1;

! Constrain factor variances

GfB-ScienceB*(v1-v6);

! Freely estimate item residual variances

RDEXP-SCINT*;

! Restrict one factor mean

[SayB*](m1);

! Restrict factor covariances to zero

GfB-ScienceB WITH GfB-ScienceB@0;

! Estimate item intercepts

! They are constrained to equality across countries

! in this model.

[RDEXP-SCINT*];

! Relations to student achievement

! Teacher aspects and general factor as predictors

! Note: InterB was excluded due to non-convergence

! of the model with InterB.

RDACH MATACH SCACH ON GfB-EasyUndB;

! Subject domain factors as predictors

RDACH ON ReadB;

MATACH ON ReadB;

SCACH ON ScienceB ReadB;

OUTPUT: stdyx;
